# Mycotoxin Determination and Occurrence in Pseudo-Cereals Intended for Food and Feed: A Review

**DOI:** 10.3390/toxins15060379

**Published:** 2023-06-04

**Authors:** María Vanessa Vila-López, Noelia Pallarés, Emilia Ferrer, Josefa Tolosa

**Affiliations:** Preventive Medicine and Public Health, Food Science, Toxicology and Forensic Medicine Department, Faculty of Pharmacy, University of Valencia, Avda. Vicent Andrés Estellés, s/n, 46100 Burjassot, Spain; mavilo@alumni.uv.es (M.V.V.-L.); josefa.tolosa@uv.es (J.T.)

**Keywords:** mycotoxins, pseudo-cereals, risk assessment

## Abstract

Nowadays, pseudo-cereals’ consumption is increasing due to their health benefits as they possess an excellent nutrient profile. Whole pseudo-cereal grains are rich in a wide range of compounds, namely flavonoids, phenolic acids, fatty acids, and vitamins with known beneficial effects on human and animal health. Mycotoxins are common contaminants in cereals and by-products; however, the study of their natural occurrence in pseudo-cereals is currently scarce. Pseudo-cereals are similar to cereal grains; thus, mycotoxin contamination is expected to occur in pseudo-cereals. Indeed, mycotoxin-producing fungi have been reported in these matrices and, consequently, mycotoxin contents have been reported too, especially in buckwheat samples, where ochratoxin A and deoxynivalenol reached levels up to 1.79 μg/kg and 580 μg/kg, respectively. In comparison to cereal contamination, mycotoxin levels detected in pseudo-cereal samples are lower; however, more studies are necessary in order to describe the mycotoxin pattern in these samples and to establish maximum levels that ensure human and animal health protection. In this review, mycotoxin occurrence in pseudo-cereal samples as well as the main extraction methods and analytical techniques to determine them are described, showing that mycotoxins can be present in pseudo-cereal samples and that the most employed techniques for their determination are liquid and gas chromatography coupled to different detectors.

## 1. Introduction

### 1.1. Pseudo-Cereals for Food and Feed

Cereals are considered as a staple food in the diet, standing at the base of the food pyramid. Currently, their consumption is relatively high, and they are commonly included as feed ingredients. However, nowadays, consumers are more focused on healthy lifestyles and appropriate nutritional habits, and, on the other hand, more attention is paid to feed composition, especially in feedstuffs for pets. Thus, due to the excellent nutrient profile of pseudo-cereals (see below), their intake is currently increasing as an alternative to cereal consumption to meet the actual requirements of the target population for both human and animal consumption [1].

Botanically, pseudo-cereals are neither grasses nor true cereal grains, but they produce fruits or seeds, which are used and consumed as cereal grains. More concretely, pseudo-cereals are dicotyledonous grains and since they produce starch-rich seeds such as cereals, they have been called pseudo-cereals [1,2]. The most common pseudo-cereals used extensively worldwide are buckwheat (*Fagopyrum esculentum*; family *Polygonaceae*), amaranth (*Amaranthus hypochondriacus*, *Amaranthus caudatus* and *Amaranthus cruentus*; family *Amaranthceae*), and quinoa (*Chenopodium quinoa* sub sp. quinoa; family *Chenopodiaceae*) [3]. These species are originally from the Andean region, but they can be cultivated worldwide as they are able to grow in adverse environmental conditions and they do not have special agronomic requirements and can be grown by simple methods [2,3,4,5]. Moreover, they offer food products with innovative nutritional qualities to meet global nutritional demands, thereby reaching various goals of the United Nations’ (UN) Agenda 2030 (https://www.un.org/sustainabledevelopment/sustainable-development-goals/ accessed on 31 January 2023), including the eradication of hunger, achieving food security, enhancing nutrition, and supporting sustainable agriculture [6,7].

Pseudo-cereals present an exceptional nutritional, phytochemical, and phenolic profile with good quality proteins. In fact, the amino acid profile and nutritional properties of pseudo-cereals in terms of essential amino acid index, biological value, protein efficiency ratio, and nutritional index are higher as compared to conventional cereals such as wheat, rice, and maize. In addition, pseudo-cereal grains show a high protein value, with an important content of lysine, an essential amino acid that is commonly the limiting amino acid in other cereals [8]. Moreover, the contents of lipids and minerals, such as calcium, magnesium, iron, potassium, and zinc, are higher than those of traditional cereals. For this reason, the interest in and consumption of pseudo-cereals as an alternative to cereals are increasing due to their nutritional properties, promoted by their beneficial effects on human health including the prevention and reduction of many degenerative diseases [1,6].

It must be highlighted that, due to the lack of gluten, these grains can be incorporated into the diet of people suffering from celiac disease, and due to their high content of starch, pseudo-cereals can be used like cereals in the preparation of value-added food products for celiac patients [1,3]. The availability of tasty gluten-free pseudo-cereals would also represent a step forward in ensuring that people with celiac disease are acquiring enough nutrients. In addition, pseudo-cereals have the potential to be included in a variety of processed functional foods since they contain bioactive compounds. Therefore, the commercialization of pseudo-cereals as functional foods would benefit people with a variety of lifestyle disorders in addition to those suffering from celiac disease.

From the processing point of view, it has been described that both buckwheat and quinoa must undergo dehulling or polishing to get rid of the saponins that give them the bitter flavor and then washing and drying prior to consumption [1,9].

Regarding animal nutrition, the inclusion of pseudo-cereal grains in feedstuffs has been increasing in the last few years, mainly due to the health benefits for different animal species. The most employed pseudo-cereal as a feedstuff ingredient is quinoa, followed by amaranth, which is being increasingly used in animal nutrition [10].

Quinoa has been used by the natives of South America since ancient times to feed ruminants and non-ruminant animals, such as cattle, sheep, horses, and pigs [11]. Although there is little research, quinoa supplementation is thought to have a high nutritional value as feed intended for ruminants [12]. In this sense, quinoa has been included as an ingredient in feed for ruminants and it has been demonstrated that it can replace clover hay in the diet by up to 45%, according to the research recently published by Ebeid et al. [13].

According to certain studies, quinoa can be ensiled to become a valuable fodder crop for dairy farms, resulting in good milk yield and greater dietary protein. Moreover, it has been reported that quinoa hay can be utilized in beef cattle feed when combined with other roughages and some chemicals. Thus, this plant may be crucial in ensuring that animal feed contains sufficient levels of macro- and microminerals. However, there are few studies on the use of quinoa in animal nutrition, and more thorough research on its application in animal feeding is required [5,14]. On the other hand, in non-ruminant nutrition, quinoa has been used especially in poultry feeding due to its content of protein and energy, thus supposing an alternative to corn [15].

Amaranth can be utilized as a grain and as fresh, dried, or ensiled forage for different animal species, mainly cattle, chickens, pigs, and rabbits. However, compared to its grain, amaranth forage has drawn a lot less study attention. Amaranth grain is classed as a growth-inhibiting grain due to the presence of heat-labile substances such as tannins, saponins, lectins, and trypsin inhibitors. Thus, due to the presence of certain anti-nutritional elements, some species of amaranth forage may not be suitable as ruminant feed or may need to undergo special processing before they can be used by ruminants. Additionally, the acceptance and use of amaranth grain by poultry and other monogastric animals are restricted by its known antinutritional properties [16]. In diets for monogastric animals, amaranth has shown good potential as an alternative ingredient, as it has been demonstrated that amaranth can improve the performance and health status of monogastric animals because of its high nutritional value and availability of phenolic compounds without having any adverse effect on animals’ productivity. Therefore, it can be said that amaranth leaves and grains can be successfully utilized in monogastric animals with the use of various processing techniques that may need to be applied in order to eliminate anti-nutritional components before use in animals [17].

In the field of companion animals, the use of pseudocereals, especially quinoa, as sources of carbohydrates is increasing due to the concern of owners for the health of their pets, comparing their dietary habits with humans on many occasions. In fact, most of the new feeds that are produced and marketed today are grain free (GF) [18,19].

Despite all this, there is hardly any research on the effect that the use of these ingredients as sources of carbohydrates has on the health of dogs and cats [20], and the few studies that have been published are limited to investigating digestibility and behavior in extrusion for the preparation of kibbles.

Regarding the possible benefits that have been observed when including pseudo-cereals in feed composition, it has been observed that the incorporation of pseudo-cereals such as quinoa and amaranth in amounts equal to or greater than 40% can be accepted by dogs without altering or negatively affecting their digestibility [20,21]. In fact, it seems that, in these cases, beneficial modifications can be produced in the fecal microbiota of dogs fed with pseudo-cereals such as the above-mentioned ones. In addition, with some pseudo-cereals, there have been increases in butyrate-producing bacteria at the intestinal level and a decrease in bacteria from the *Fusobacteriaceae* family when compared to feeds made with rice [20]. Regarding the behavior in extrusion, if feeds made with GF grains are compared with those made with pseudo-cereals, it seems that there are significant changes, as well as in the palatability of the feed before adding the palatizer [21].

### 1.2. Major Mycotoxins in Pseudo-Cereals

Like cereals and oilseeds, pseudo-cereal grains are susceptible to fungal growth and mycotoxin contamination; however, these matrices have received little attention in the literature, especially compared to cereal grains [22], although some studies have demonstrated the actual risk of pseudo-cereal contamination by mycotoxins, as indicated in Section 2.

Mycotoxins are toxic metabolites produced by different fungal species or strains within the same fungal species during secondary fungal metabolism. The main hazardous mycotoxins reported are aflatoxins (AFs) (mainly produced by *Aspergillus*), fumonisins (FBs), zearalenone (ZEA), and trichothecenes (TCs) (produced by *Fusarium*), and ochratoxins (OTs) and patulin (PAT) (mainly produced by *Penicillium*) [23,24].

Mycotoxins have been associated with a broad range of toxic effects on both humans and animals, depending on their different chemical structures, including acute toxicity, hepatotoxicity, nephrotoxicity, genotoxicity, carcinogenicity, immunotoxicity, mutagenicity, teratogenicity, and reproductive toxicity [25,26]. Regarding their carcinogenic potential, their human carcinogenicity risk has been assessed by the International Agency for Research on Cancer (IARC), classifying AFs as carcinogenic substances in category “1” while FB1 and FB2 are classified in group “2B” as possible carcinogenic substances to humans [23,27].

Due to the above-mentioned adverse effects, some mycotoxins have been recognized by European regulatory authorities as emerging risks in food safety regarding animal and human health, thus, efficient regulatory frameworks and monitoring approaches have been developed globally relying on the latest scientific knowledge based mainly on new analytical tools and approaches. In this sense, maximum levels (MLs) have been established for the main classes of mycotoxins in several core commodities intended for food and feed by the European Union (EU), including cereals, nuts, fruits, and derived products, such as milk. Those MLs are compiled in EU Commission Regulation (EC) No 1881/2006 [28] and its amendments. However, different drawbacks exist nowadays, as the current MLs do not consider the exposure to multiple mycotoxins, and they are either based on the risk assessment of a single compound or their sum and no MLs have been established for pseudo-cereal grains [29,30,31].

Due to the scarce information on mycotoxin occurrence in these matrices, this manuscript aims to review the existing literature on the natural occurrence of mycotoxins in pseudo-cereals intended for both food and feed and the most common extraction and analytical methods employed for their determination.

## 2. Occurrence and Co-Occurrence of Mycotoxins in Pseudo-Cereal Samples for Food and Feed

Although it has been reported that pseudo-cereal grains can be as susceptible to fungal growth and mycotoxin contamination as cereal grains, there is still scarce information on the contaminating fungi and mycotoxin occurrence in these matrices. Some drawbacks can be found when searching for information on mycotoxin occurrence in pseudo-cereal grains. On the one hand, the few available studies focused primarily on the investigation of the mycoflora present in pseudo-cereal grains and not on mycotoxin contamination. On the other hand, another problem when searching mycotoxin data on pseudo-cereal grains in the literature is that this information is usually mixed together with cereal data; thus, it is difficult to search these studies by the title of the manuscript or by including “pseudo-cereal” as the keyword as they are mainly referred to as cereals [32].

As previously stated, some reported surveys have been focused on the mycoflora present in pseudo-cereal grains. In this sense, some studies have reported the presence of mycotoxin-producing fungi in pseudo-cereal samples. Thus, the presence of *Ascohyta*, *Altenaria*, *Phoma*, *Fusarium*, *Bipolaris*, *Cladosporium*, and *Pyronochaeta* genera in quinoa seeds (*Chenopodium quinoa)* from Bolivia, Brazil, Czech Republic, and Peru have been reported, but no data on mycotoxin occurrence were provided in these studies [31]. Some of these fungal genera were also found by Krysińska-Traczyk et al. (2007) [33] in buckwheat grain and buckwheat grain dust, where *Penicillium* spp., *Mucor mucedo*, *Alternaria alternata*, and *Cladosporium lignicola* were the predominant genera in buckwheat grain, while *Rhodotorula rubra*, *Mucor mucedo*, *Alternaria alternata*, and *Penicillium* spp. were more abundant in buckwheat grain dust. Regarding the *Fusarium* strains, *F. culmorum* was recovered from samples of grain dust but it was not detected in grain samples.

In the survey conducted by Pappier et al. (2008) [34], the authors reported *Penicillium* and *Aspergillus* as the most frequently encountered fungal genera in quinoa samples from Argentina. Interestingly, these authors reported a reduction in *Aspergillus* incidence when processing the grains for the removal of saponins; however, the incidence of *Penicillium*, *Eurotium*, *Mucor*, and *Rhizopus* increased after the treatment. These results agree with those reported by Bresler et al. (1995) [35] who found that the most prevalent mycoflora in amaranth grains from Argentina corresponded to the mycotoxin-producing fungal species *Aspergillus flavus* (*A. flavus*), *A. parasiticus*, *Penicillium chrysogenum*, and *Fusarium equiseti* (*F. equiseti*). *Penicillium* spp., *Fusarium* spp., and *Aspergillus* spp. were also the predominant mycoflora reported by Ramos-Díaz et al. (2021) in pseudo-cereal samples [31].

According to the study carried out by Vásquez-Ocmín et al. (2022) [36] in pseudo-cereal samples from Peru, there were several mycotoxigenic fungi detected, namely *Aspergillus* spp., *Penicillium* spp., and *Alternaria* spp.; however, *Fusarium* spp. was not detected. On the contrary, *Fusarium* spp. was detected in 92% of the amaranth grain from Peru analyzed by Ducos et al. (2021) [37], while in the study reported by Sacco et al. (2020) [24], *Aspergillus* spp. was detected in amaranth, buckwheat, and quinoa samples and *Penicillium* spp. was detected in buckwheat and quinoa samples.

Regarding the most common mycotoxigenic fungal species, *Penicillium* spp. and *Alternaria* spp. were the most frequently detected in buckwheat samples. In quinoa samples, *Penicillium* spp. and *Aspergillus* spp. were the most reported fungal species while in amaranth grains, *Aspergillus* spp., *Penicillium* spp., and *Fusarium* spp. were detected, highlighting the contamination by *Fusarium* species.

Although a very limited number of studies have measured mycotoxins in pseudo-cereal grains, especially compared to cereals, in Table 1 it can be observed that mycotoxin occurrence in these matrices has been reported in different surveys. Original research papers as well as reviews have been included in this section, regardless of the year of publication, as all searches were performed without any time restriction.

The most reported mycotoxins in pseudo-cereal samples are those produced by *Fusarium* spp., such as TCs, FBs, ZEA, and the so-called emerging *Fusarium* mycotoxins, such as enniatins (ENNs) and beauvericin (BEA).

Among TCs, DON and T-2 toxins have been widely reported to occur mainly in buckwheat samples, showing higher incidences and contents for DON in this matrix [32,33,43].

FBs were reported at high levels in pseudo-cereal samples from Italy [24], with the highest contents (up to 567.25 μg/kg) corresponding to buckwheat samples.

In the survey conducted by Bresler et al. (1991) [38], ZEA was determined among the analyzed mycotoxins at worrying levels of up to 1980 μg/kg and 420 μg/kg in two samples of amaranth grains (*Amaranthus cruentus*). Later, these authors showed that amaranth grains possess certain susceptibility to ZEA contamination by incubation of amaranth seeds with *F.equiseti* [48]. ZEA was also reported to occur in buckwheat products as indicated by Kirinčič et al., 2015 [32]. However, neither ZEA nor TCs were detected in amaranth, quinoa, and buckwheat samples from Germany according to Schollenberger et al., 2005 [39], although several TCs were included in the survey, namely diacetoxyscirpenol (DAS), 15-monoacetoxyscirpenol (MAS), scirpentriol (SCIRP), T-2, HT-2 toxin (HT-2), T-2 triol, T-2 tetraol, neosolaniol (NEO), DON, 3- and 15-acetyl-DON (3-, 15-acDON), nivalenol (NIV), and fusarenon-X (FUS-X). Regarding cereal contamination, wheat has been reported to contain high DON levels, with mean concentrations up to 1025.4 μg/kg, and NIV with mean levels up to 75.2 μg/kg [30].

Other *Fusarium* mycotoxins such as ENNs and BEA have been widely reported in cereal samples even at high levels [49,50,51]. In the study reported by Uhlig et al. (2006) [49] ENNs and BEA were reported to occur in cereal samples showing high incidences and levels. ENNB showed the highest prevalence (100%) and the highest maximum concentration (5800 μg/kg) in wheat samples. Fortunately, lower contents have been reported in pseudo-cereals. In pseudo-cereals, ENNs have been detected in quinoa samples according to the study reported by Besaire et al. (2021) [47]. Although BEA was not detected in that survey, levels up to 7.9 μg/kg were detected according to Vásquez-Ocmín et al. (2022) [36]. Regarding food safety, these authors pointed out that the natural occurrence of BEA in quinoa grains originating from the Cajamarca region represents a point of concern, due to the promotion of quinoa cultivation in this region by the local authorities [36].

Due to their adverse toxic effects, AFs have been surveyed in several studies. Although a major number of the studies did not find AFs levels in pseudo-cereal samples [32,34,36,38,40,41,46,47], some studies revealed AFs’ occurrence in these matrices, and some studies reported the absence of AFs in buckwheat samples but detected AFs contents in cereal samples, such as rye (0.01 μg/kg) and rice (1.06 μg/kg). Approximately 100% of buckwheat samples were contaminated with AFB1 as reported by Keriene et al. (2016) [43,44]. It must be highlighted that raw buckwheat hulls (without steamed treatment) showed higher AFB1 contents (75.8 μg/kg) than the grain [44], showing a protective effect of the hull over the inner part as demonstrated by other researchers in nut samples [52]. Interestingly, these authors reported that the samples with higher concentrations of AFB1 had significantly (*p* < 0.05) lower concentrations of DON, thus suggesting possible interaction between fungal species producing AFs and TCs [44].

AFB1 was also detected in the Tartary buckwheat seeds in the study reported by Ren et al. (2018) [45] and in buckwheat samples as reported by Sacco et al. (2020) [24]. Similar AFs levels have been reported in quinoa samples in surveys reported by Sacco et al. (2020) [24] and by Ramos-Díaz et al. (2021) [31], while lower contents were found in amaranth seeds in those studies [10,18]. These authors reported varying mycotoxin levels depending on the type of crop, geographical location, and agricultural practices used. Differences between regions have been observed by these authors regarding mycotoxin occurrence in pseudo-cereals and cereals. In general, pseudo-cereal grains from North Europe showed higher mycotoxin contamination than those from South America, while the opposite occurred with cereal grains [31].

Regarding OTA occurrence in pseudo-cereal grain samples, only two studies did not find this compound in quinoa and amaranth samples [36,38], while other surveys found OTA as a common mycotoxin contaminating pseudo-cereal samples. In the study conducted by Sugita-Konishi et al. (2006) [40], OTA was found in buckwheat flour and dried buckwheat noodles with contents up to 1.79 μg/kg, and similar contents were detected in this study in rye (2.59 μg/kg), while lower contents were detected in wheat flour (0.48 μg/kg) and oatmeal (0.18). These results are in accordance with those reported by Krysińska-Traczyk et al. (2007) [33], Kumagai et al. (2008) [41], and Kirinčič et al. (2015) [32], as all of them reported similar OTA contents in buckwheat samples of different origin. However, lower OTA levels were found by other researchers also in buckwheat from Lithuania and Serbia [43,44,46]. It must be highlighted that these authors also found lower levels of OTA in cereal samples, such as wheat (up to 0.40 μg/kg), barley (up to 0.11 μg/kg), and oat (up to 0.09 μg/kg); however, higher contents were detected in rye samples (up to 23 μg/kg) [46].

As in cereal samples, differences have been reported regarding mycotoxin levels in pseudo-cereal grains from organic agriculture. Thus, higher mycotoxin contents were reported by Sacco et al., 2020 [24] and Keriene et al., 2016 [43] in organic buckwheat samples.

In the study carried out by Herrera et al. (2019) [53], gluten-free cereal samples for infants under six months were analyzed, some of them including quinoa and buckwheat. The results showed that ten samples were positive for at least one mycotoxin (four for AFs and six for DON). This study found that AFB1 and AFG1 co-occurred in five samples, and one sample based on rice and quinoa contained AFB2, AFG1, and AFG2 but not AFB1. Moreover, 8 samples out of 14 samples of multi-cereals for infants above 6 months were positive for AFs, while 6 samples out of these 14 were positive for DON contamination.

## 3. Mycotoxin Determination

Many techniques have been approved and used for the analysis of mycotoxins in food and feed since the first mycotoxins were discovered. Despite the significant advancements made in this area, there are still several obstacles and shortcomings with these analytical techniques that need to be resolved [54]. The enormous variety of mycotoxin chemical structures, the co-occurrence of mycotoxins, problems in detecting low-level mycotoxin contamination, complex food matrices where the mycotoxin contamination occurs, and difficult extraction techniques are just a few of the analytical issues [55]. Continuous improvements in the analytical methodology for mycotoxin analysis in a variety of food matrices are required to address these issues in order to support the enforcement of mycotoxin regulations, safeguard consumer health, promote the agriculture sector, and facilitate global food trade [54].

Both the extraction methods and analytical equipment used for mycotoxin determination in pseudo-cereal samples are the same or very similar to those employed for cereal matrices, as they show a similar composition. In the next sections, the most employed techniques to extract and determine different mycotoxins in pseudo-cereal samples are described.

### 3.1. Extraction Methods

Given that pseudo-cereals are extremely complex food matrices, extraction to isolate the target chemicals and purification or clean-up to remove impurities must always be carried out before analysis. Moreover, due to the imposed legal constraints, mycotoxins are typically found naturally in extremely low concentrations in food. In order to facilitate the subsequent detection of the analyte, it would be necessary to perform a concentration process in addition to the other steps [56,57].

The most common methodology used for mycotoxin extraction is solid–liquid extraction (SLE) followed by a purification or clean-up step when necessary, with the most widely used technique being solid-phase extraction (SPE), mainly with C18, or immunoaffinity columns (IACs), which contain specific antibodies to the analyte of interest [57,58]. However, IACs are costly and intricate purification systems, their utility in multiclass analysis is constrained by their high selectivity, and they also suffer from low recoveries for some mycotoxins. Therefore, multiclass extraction methods that are easier to use, more effective, and environmentally beneficial are needed. Among the different strategies, the so-called QuEChERS (Quick, Easy, Cheap, Effective, Rugged, and Safe), which is a type of dispersive SPE (dSPE) used for sample preparation, is commonly used for the extraction of a wide range of mycotoxins in different food matrices (Table 2) [22]. It consists of two steps: (i) extraction based on partitioning by salting-out, which involves the equilibrium between an aqueous and organic layer, and (ii) dSPE extraction for additional clean-up utilizing a mix of MgSO_4_ and other sorbents, such as C18 or primary and secondary amine (PSA) [22,59].

On the other hand, in a conventional SLE, a compound or a group of compounds that are a part of a solid are dissolved in a solvent with the appropriate polarity. Most of the mycotoxins in this situation can be effectively extracted using comparatively polar solvents such as acetonitrile (MeCN), acetone, or methanol (MeOH) [60]. Additionally, it is practical to use small quantities of acidified water to moisten the solid to improve the efficiency of the extraction. By doing this, the latter’s contact surface with the solvent can be increased, and the pH is made more acidic, which enhances the mycotoxins’ solubility [61]. Since this kind of extraction is not very specific, it is frequently followed by purification or clean-up to get rid of any interfering substances that can affect the analysis’s outcomes, such as lipids, proteins, and coloring agents. Chelating chemicals (C18 and PSA) can be used to eliminate them in significant quantities [58,61].

### 3.2. Analytical Determination

Among the different analytical tools for mycotoxin determination, the most commonly used include thin-layer chromatography (TLC), liquid chromatography (LC), high-performance liquid chromatography (HPLC), and gas chromatography (GC) (Table 2) [62]. For quantification purposes, LC and GC can be coupled to different detectors, such as ultraviolet (UV), fluorescence (FL), or mass spectrometry (MS) [22]. Initially, UV and FL were widely used for mycotoxin determination [32,39,40,46]. These detection techniques are based on the UV absorbance and fluorescence characteristics of mycotoxins. Generally, the sensitivity of fluorescence is 10–1000 times higher than the sensitivity of the UV detector, so FL is used conventionally in the determination of specific fluorescent compounds present in cereal and pseudo-cereal samples. FL detection has been widely used in the analysis of AFs, OTA, and ZEA in cereals and pseudo-cereals matrices with good accuracy and high precision [63]. For instance, Torović (2018) [46] analyzed the presence of AFs and OTA in samples of wheat, buckwheat, rye, oat, barley, rice, millet, and corn flour employing HPLC with FL detection. The analytical method developed showed good performance, with recoveries ranging from 53.1% to 85.0% for AFB1 and from 70.3% to 88.3% for OTA and limits of detection (LODs) and quantification (LOQ) of 0.04/0.1 μg/Kg for AFB1 and 0.07/0.2 μg/Kg for OTA, respectively.

However, nowadays, LC or HPLC coupled to tandem mass spectrometry detectors (LC–MS/MS) is the most widely used techniques as it allows one to obtain an accurate and reliable determination of several mycotoxins at even low concentrations in complex matrices. Moreover, it allows the simultaneous determination of mycotoxins belonging to different chemical families [64]. In this sense, Ramos-Diaz et al. [31] developed a multi-analyte LC–MS/MS method to detect mycotoxins and fungal metabolites in pseudo-cereal grains (quinoa and kăniwa). The study documented the detection of 101 analytes at varying levels with recoveries ranging from 70 to 120% (Table 2). Moreover, Veršilovskis et al. (2008) [42] previously developed a sensitive LC-MS/MS method for the analysis of STC in buckwheat and other cereal grains. This method included sample extraction with acetonitrile/water solution followed by SPE.

Another tool for mycotoxin determination commonly employed is ELISA, which consists of an immunological assay for mycotoxin screening [24,44]. This assay provides fast and economical measurements and does not require previous clean-up procedures. ELISA formats (direct, indirect, competitive, and sandwich) are recognized as adequate for mycotoxin screening. To favor field analysis, a transduction system with appropriate molecular recognition elements has been integrated. In this sense, different analytical methods have been developed for AFs, OTA, FB1, and TCs’ detection in cereals and pseudo-cereals (Table 2) [63]. However, the number of matrices tested is limited, and at low concentrations, method precision can be reduced. In addition, the presence of matrix interferents and structurally related mycotoxins can alter antibody binding and, subsequently, mycotoxin quantification. For instance, in buckwheat groats, Keriene et al. [44] reported limits of detection (LODs) for ELISA determination of 3.5 μg/Kg for T-2, 2.5 μg/Kg for OTA, and 1 μg/Kg for AFB1. According to Sacco et al. (2020) [24], who used an ELISA for AFs and FBs determination, in this case, the ELISA detection range was 2–200 μg/Kg for AFB1, 1.75–140 μg/Kg for total AFs, and 222–6000 μg/Kg for FBs.

Although ELISA and UV and FL detectors have been widely employed, nowadays, the most employed technique is ultra-high-performance liquid chromatography coupled to mass spectrometry, commonly used for quantitative multi-mycotoxin analysis. In general, a low-resolution mass spectrometer (MS) is used for analysis or simply for compound identification purposes; however, it may not be enough to distinguish between two molecules with the same molecular mass. In this sense, HRMS constitutes a more sensitive and accurate detector able to distinguish between two substances with similar masses with a high level of certainty and confidence.

In the studies carried out by Bessaire et al. (2021) [47] and Arroyo-Manzanares et al. (2014) [22], HRMS and MS/MS techniques, respectively, were combined with QuEChERS extraction, while in other surveys, they have been combined with other novel extraction methods. Thus, Bochetto et al. (2021) [8] used an extraction/preconcentration procedure that consisted of in-phase liquid–liquid microextraction based on the solidification of a floating organic drop followed by double solvent-assisted back-extraction (DLLME-SFO-SBE) followed by UHPLC-MS/MS determination, while Vásquez-Ocmín et al. (2022) [36] used biphasic microextraction. These extraction techniques allowed one to extract even small mycotoxin amounts which can be detected and quantified due to the high specificity and sensitivity of the MS/MS and HRMS detectors. Both extraction methods allow one to reduce the solvent amount used during the extraction step; thus, the future trend in mycotoxin extraction is to develop green alternatives to conventional methods.

## 4. Conclusions

Mycotoxins are common chemical contaminants present mainly in cereal products and their by-products. Nowadays, in both human and animal nutrition, nutritional habits are changing towards healthier ingredient consumption; thus, in some cases, cereals are being replaced by pseudo-cereal grains, such as quinoa, amaranth, and buckwheat, due to their multifarious health benefits. These beneficial effects are due to the presence in their composition of flavonoids, phenolic acids, fatty acids, vitamins, dietary fibers, minerals, and other bioactive compounds that are present in pseudo-cereals. As cereals and pseudo-cereals are similar, it is expected for mycotoxin contamination to be found in pseudo-cereal samples; however, the investigation of their natural occurrence in these matrices is still scarce. Despite this, some studies have reported the occurrence of some mycotoxins, such as AFs, OTA, FBs, TCs, and ZEA, among others, in quinoa, amaranth, and buckwheat samples. The extraction methods and analytical tools employed for mycotoxin determination in pseudo-cereal samples are the same employed for cereal analysis, with SLE followed by SPE or clean-up being the most employed extraction techniques and LC or GC coupled to different detectors, mainly UV, FL, and MS/MS, being the most employed analytical equipment.

## Figures and Tables

**Table 1:** Mycotoxin occurrence in different pseudo-cereal samples.

Pseudocereal Sample	Detected Mycotoxins	Incidence (%) *	Mycotoxin Contents Media ± SD ** (Range) μg/kg	Reference
Amaranth	ZEA AFs OTA STG	100 nd nd nd	(420.0–1980.0) nd nd nd	[38]
Amaranth, quinoa, and buckwheat	TCs ZEA, α-ZOL, and β-ZOL	nd nd	nd nd	[39]
Buckwheat flour and dried buckwheat noodles, among others	AFs OTA FBs	nd 60 nd	nd 0.51 (0.16–1.79) nd	[40]
Buckwheat grain Buckwheat grain dust	DON OTA DON OTA	33.3 50 100 100	(74.0–87.0) (0.38–1.14) (10.0–283.0) (1.03–2.42)	[33]
Buckwheat flour and dried buckwheat noodles, among others	AFs OTA	nd 80	nd 0.51 (0.16–1.79)	[41]
Quinoa	AFs CIT CPA OTA	nd nd nd nd	nd nd nd nd	[34]
Buckwheat	STG	17 20	(0.5–25) (25–200)	[42]
Quinoa and Amaranth	AFs, OTA, FBs, NIV, DON, FUS-X, T-2/HT-2, CIT, STG, and ZEA	nd	nd	[22]
Buckwheat and buckwheat products	OTA DON ZEA AFs FBs T-2/HT-2	4 17 8 nd nd nd	2.1 98.0 ± 40 (max 141.0) 13.0 ± 7 (max 18.0) nd nd nd	[32]
Buckwheat grain	AFB1 DON OTA T-2	100 100 100 100	(2.18–8.39) (300.0–580.0) traces (24.0–38.0)	[43]
Buckwheat groats	AFB1 OTA T-2	100 100 100	(2.3 ± 0.6) (0.7 ± 0.2) (4.1 ± 0.5)	[44]
Tartary buckwheat seeds	AFB1	7	5.62	[45]
Buckwheat flour	OTA AFs	38.5 nd	0.40 ± 0.34 (0.15–0.96) nd	[46]
Quinoa Amaranth (grain) Buckwheat	AFs FB1 + FB2 AFs FB1 + FB2 AFs FB1 + FB2	nr nr nr nr nr nr	4.4 ± 0.8 368.5 ± 2.5 1.60 ± 0.70 111.00 ± 0.0 4.68 ± 0.44 567.25 ± 138.14	[24]
Quinoa	AFB1 BEA DON ENNA ENNA1 ENNB ENNB1 Mycophenolic acid Tentoxin Tenuazonic acid	nd nd nd ***id ***id ***id ***id nd nd ***id	- - - nr nr nr nr - - nr	[47]
Quinoa	AFB1 FB1 OTA PAT BEA	nd 32 nd nd 82	nd traces nd nd 7.9 ± 0.85	[36]

AFB1: aflatoxin B1; AFs: aflatoxins; BEA: beauvericin; CIT: citrinin; CPA: cyclopiazonic acid; DON: deoxynivalenol; ENNA: enniatin A; ENNA1: enniatin A1; ENNB: enniatin B; ENNB1: enniatin B1; FB1: fumonisin B1; FB2: fumonisin B2; FBs: fumonisins; OTA: ochratoxin A; PAT: patulin; STG: sterigmatocystin; TCs: trichothecenes; T-2: T-2 toxin; HT-2: HT-2 toxin; ZEA: zearalenone; α-ZOL and β-ZOL: α—and β—zearalenol; nd: not detected; nr: not reported; * Incidence (%): positive samples from the total number of analyzed samples expressed as a percentage; ** SD: Standard deviation; ***id: compounds identified but no quantification data provided.

**Table 2:** Main extraction and analytical methods for mycotoxin determination in pseudo-cereal samples.

Samples	Mycotoxins	Extraction Method	Analytical Method	Reference
Amaranth	ZEA, AFs, OTA, STG	SLE and clean-up	TLC	[38]
Amaranth, quinoa, and buckwheat	TCs ZEA, α-ZOL, and β-ZOL	SLE and clean-up by SPE SLE and IAC	GC-MS HPLC-FLD	[39]
Buckwheat flour and dried buckwheat noodles, corn, rice, peanuts, peanut butter, popcorn, cornflakes, and sesame oil	AFs OTA FBs	SLE and IAC SLE and IAC SLE and IAC	HPLC-FLD HPLC-MS	[40]
Buckwheat grain and buckwheat grain dust	DON NIV OTA	SLE and SPE clean-up SLE and IAC	GC-MS GC-MS HPLC	[33]
Corn, processed corn, buckwheat, dry buckwheat noodles, peanuts, rice, and sesame oil	AFs OTA	SLE and IAC	HPLC	[41]
Wheat, buckwheat, barley, oats, and rye	STG	SLE and SPE clean-up	LC–MS/MS	[42]
Buckwheat, quinoa, spelt, amaranth, and white rice	AFs, FBs, OTA, T-2, HT-2, STG, CIT, ZEA, NIV, DON, and FUS-X	QuEChERS	UHPLC-MS/MS	[22]
Buckwheat and buckwheat products, wheat, maize, oat, rice, rye, barley, millet, triticale, and others	OTA DON ZEA AFs FBs T-2/HT-2	SLE and IAC	HPLC-FLD HPLC-DAD HPLC-FLD LC-MS/MS LC-MS/MS GC-MS	[32]
Buckwheat groats	AFB1 OTA T-2	-	ELISA	[44]
Tartary buckwheat seeds	AFs, FBs, OTA, ZEA, DON, and T-2/HT-2	SLE	UFLC-QTrap-MS/MS	[45]
Wheat, buckwheat, rye, oat, barley, rice, millet, and corn flour	OTA AFs	SLE and IAC	HPLC-FLD HPLC-FLD	[46]
Quinoa, amaranth (grain), and buckwheat	Afs FB1 + FB2	-	ELISA	[24]
Pea protein, soy protein, red quinoa, and wheat flour	DON, AFs, ZEA, DON, FBs, ENNA, HT-2, OTA, PAT, and T-2	QuEChERS	UHPLC-MS/MS	[47]
Corn, wheat, amaranth, rice, barley, and oats	FBs, DON, and ZEA	SLE	HPLC-MS/MS	[37]
Quinoa, kăniwa, barley, oat, and wheat	101 mycotoxins	SLE	LC-MS/MS	[31]
Quinoa	AFB1, FB1, OTA, PAT, and BEA	SLE	UHPLC−HRMS	[36]
Amaranth	DON and ZEA	In-phase liquid–liquid microextraction based on the solidification of a floating organic drop followed by double solvent-assisted back-extraction (DLLME-SFO-SBE)	UHPLC-MS/MS	[8]

AFs: aflatoxins; CIT: citrinin; DON: deoxynivalenol; ELISA: enzyme-linked immuno sorbent assay; ENNA: enniatin A; FLD: fluorescence detector; FBs: fumonisins; GC-MS: gas chromatography/mass spectrometry; HPLC: high-performance liquid chromatography; HPLC-DAD: high-performance liquid chromatography-diode array detector; HPLC-FLD: high-performance liquid chromatography-fluorescence detection; HPLC-MS: high-performance liquid chromatography-mass spectrometry; HPTLC: high-performance thin-layer chromatography; HPLC-UV: high-performance liquid chromatography-ultraviolet detection; HT-2: HT-2 toxin; IAC: immuno affinity columns; LC-MS: liquid chromatography-mass spectrometry; MS/MS: tandem mass spectrometry; NIV: nivalenol; OTA: ochratoxin A; PAT: patulin; QuEChERS: Quick, Easy, Cheap, Effective, Rugged, and Safe; S-L extraction: solid–liquid extraction; STG: sterigmatocystin; T-2: T-2 toxin; UHPLC−HRMS: ultra-high-performance liquid chromatography high-resolution mass spectrometry; UFLC-QTrap-MS/MS: ultra-fast liquid chromatography coupled with triple quadrupole mass spectrometry; ZEA: zearalenone.

## Data Availability

The data presented in this study are available in this article.
